# 自体造血干细胞移植治疗伴重度肾功能不全新诊断多发性骨髓瘤5例报告并文献复习

**DOI:** 10.3760/cma.j.issn.0253-2727.2023.07.012

**Published:** 2023-07

**Authors:** 云 鲁, 广宁 石, 顺宗 袁, 秀斌 肖, 喜林 陈, 艺 马, 世华 赵, 俊丽 陈, 雪利 张, 玥琦 王, 文荣 黄

**Affiliations:** 解放军总医院第五医学中心血液病医学部血液科，北京 100071 Department of Hematology, the Fifth Medical Center of Chinese PLA General Hospital, Beijing 100071, China

自体造血干细胞移植（auto-HSCT）是多发性骨髓瘤（MM）患者的一线治疗方法[1]，已在国内得到广泛应用。肾功能不全在初治MM患者中发生率高达30％～40％，部分重症患者需要血液透析治疗[2]。重度肾功能不全MM患者行auto-HSCT的安全性和疗效越来越受到国内外临床医师重视，国内尚无相关报道。我中心近期应用auto-HSCT治疗5例重度肾功能不全MM患者并获得较好的临床疗效。

## 病例与方法

一、病例资料

本研究纳入2021年9月至2022年3月在我中心完成auto-HSCT的5例伴重度肾功能不全MM患者，对其临床资料进行回顾性分析。纳入标准：①符合2022年中国多发性骨髓瘤诊治指南诊断标准[3]；②auto-HSCT时合并重度肾功能不全，肾小球滤过率（eGFR）<30 ml/min/1.73 m^2^）。

二、预处理方案

美法仑（Mel）200 mg/m^2^或140 mg/m^2^。

三、支持治疗

患者移植期间在层流病房行全环境保护。预处理前常规给予左氧氟沙星口服1周，同时口服阿昔洛韦预防疱疹病毒感染、复方磺胺甲恶唑预防卡氏肺孢子虫感染、氟康唑胶囊预防真菌感染。除1例透析依赖MM患者外，其余患者均在移植后3 d给予聚乙二醇化重组人粒细胞刺激因子。血小板计数低于20×10^9^/L或有出血表现时给予辐照机采血小板输注。

四、造血重建标准

粒细胞植入为连续3 d中性粒细胞计数（ANC）>0.5×10^9^/L，血小板植入为连续7 d血小板计数>20×10^9^/L且脱离血小板输注。

五、安全性评价

移植过程中的不良反应根据WHO毒性反应分级标准判定。移植后100 d内与原发疾病无关的治疗相关死亡率定义为移植后100 d移植相关死亡率（TRM）。

六、疗效评价和随访

患者出院后采用门诊复查、电话联系等方式进行随访，随访截至时间为2022年7月31日，中位随访时间为201（150～323）d。疗效评价参照文献[3]，分为严格的完全缓解（sCR）、完全缓解（CR）、非常好的部分缓解（VGPR）、部分缓解（PR）、疾病稳定（SD）和疾病进展（PD）。肾脏疗效评价参照文献[4]。

## 结果

一、临床特征

本研究共纳入5例MM患者，病例具体特征见表1。其中男3例，女2例，中位年龄56（47～66）岁。轻链型2例，IgA-k型2例，IgG-λ型1例；修订的国际分期系统（R-ISS）分期均为ⅢB期。在移植前，5例患者的eGFR分别为25.43、14.84、27.10、16.66、26.49 ml/min/1.73m^2^，例4在移植前为透析依赖。

**表1:** 5例伴重度肾功不全多发性骨髓瘤患者自体造血干细胞移植情况及血液学疗效

例号	性别	年龄（岁）	疾病类型	美法仑剂量（mg/m^2^）	单个核细胞输注量（×10^8^/kg）	CD34^+^细胞输注量（×10^6^/kg）	粒细胞植入时间（d）	血小板植入时间（d）	并发症	随访时间（d）	移植前血液学疗效	移植后90 d血液学疗效
1	男	65	κ轻链	140	32.30	1.90	11	84	粒细胞缺乏伴发热、口腔黏膜炎	258	VGPR	CR
2	男	58	IgG-λ	140	8.37	6.53	11	14	粒细胞缺乏伴发热、口腔黏膜炎	201	CR	CR
3	女	47	IgA-κ	200	14.19	8.84	9	9	口腔黏膜炎、肠炎	150	PD	PR
4	男	66	λ轻链	140	16.15	5.38	13	10	粒细胞缺乏伴发热、口腔黏膜炎、重症肠炎、肠道感染	142	sCR	sCR
5	女	56	IgA-κ	140	10.69	3.47	10	17	粒细胞缺乏伴发热、口腔黏膜炎、心功能不全、心律失常	323	CR	sCR

注 CR：完全缓解；sCR：严格的完全缓解；PR：部分缓解；VGPR：非常好的部分缓解；PD：疾病进展。5例患者移植前疾病分期均为修订的国际分期系统（R-ISS）ⅢB期

二、造血重建

5例患者回输的单个核细胞中位数为14.19（8.37～32.3）×10^8^/kg，CD34^+^细胞中位数为5.38（1.90～8.84）×10^6^/kg。中位粒细胞植入时间为11（9～14）d，中位血小板植入时间为14（9～84）d。

三、血液学疗效

移植后100 d进行血液学疗效评估：移植前1例VGPR患者获得CR，1例PD患者达到PR、1例CR患者提升到sCR、两例维持CR和sCR疗效。

四、移植相关不良反应

主要的非血液学不良反应为胃肠道反应，2级恶心及呕吐2例、3级3例；腹泻均为2级；口腔黏膜炎3级1例、2级4例。4例患者发生粒细胞缺乏伴发热，抗感染治疗后均得到控制，中位发热持续时间为4（1～6）d。5例患者中1例出现1级肝功能损伤，余4例未出现肝功能损伤。移植后100 d内未发生移植相关死亡。

五、肾功能变化

5例患者均在美法仑输注后出现一过性急性肾损伤（AKI），表现为血肌酐一过性升高，按照IMWG急性肾损伤分级标准[5]均为1级，血肌酐峰值出现中位时间为2（1～23）d，较中位升幅32.4％（23.0％～46.5％），但在峰值后2～7 d降至移植前水平，具体见表2。透析依赖患者预处理化疗期间行2次透析，其余患者均未行透析。

移植后90 d肾脏疗效评价均为微小缓解（MR）。5例患者移植后30 d、90 d的eGFR较移植前均有不同程度改善，24小时尿蛋白也有不同程度下降。1例透析依赖患者在移植后30 d脱离透析，详见表2。

**表2:** 5例重度肾功不全多发性骨髓瘤患者自体造血干细胞移植前后肾功能变化

例号	血肌酐升幅（%）		eGFR（ml/min/1.73m^2^）				24小时尿蛋白（g）		移植后90 d肾脏疗效评价
			移植前	移植后30 d	移植后90 d		移植前	移植后90 d	
									
1	23.0		25.43	47.67	57.94		0.55	0.72	MR
2	43.7		14.84	17.24	23.98		0.5	0.4	MR
3	31.4		27.10	56.31	36.21		1.97	1.16	MR
4	46.5		16.66	20.55	23.78		0.2	0.18	MR
5	33.5		26.49	38.86	35.02		0.41	0.55	MR

注 eGFR：估算肾小球滤过率；MR：微小缓解

## 讨论

对于伴有重度肾功能不全的MM患者来说，auto-HSCT过程的安全性、肾脏耐受程度、移植后肾功能改善情况是移植最关键的问题。

本组重度肾功能不全MM患者接受大剂量美法仑预处理方案过程顺利，移植后100 d内未发生移植相关死亡。5例患者粒细胞植入时间为11（9～14）d，血小板植入时间为14（9～84）d，依赖透析患者的造血重建时间并未较其他患者延长。来自国际骨髓移植登记处1 452例MM患者分析显示，67例重度肾功能不全患者粒细胞、血小板植入时间分别为11.5（11～15）d、18（11～37）d，与中度肾功能不全及肾功能正常患者相比差异无统计学意义[2]。本组病例数据与该报道近似，其中1例患者血小板植入时间延长考虑与回输CD34^+^细胞数偏少（1.9×10^6^/kg）有关。本组病例移植不良反应发生率低于波兰骨髓瘤工作组的报道[6]，5例患者在移植过程中均有不同程度的黏膜炎、胃肠道反应和感染，5例患者均未发生3级以上黏膜反应，粒细胞缺乏伴发热很快得到控制，可能与积极对症支持治疗有关。

本研究中3例患者auto-HSCT后血液学疗效进一步改善，5例患者进行脏器功能评估证实肾脏功能均达到MR且透析患者脱离透析。证实通过auto-HSCT可以改善肾功能不全MM患者的疗效及肾脏功能。Andronesi等[7]报道了185例行auto-HSCT的MM患者，19例（10.3％）患者在移植后发生AKI，其中1级18例（9.7％），2级1例（0.6％），均在3个月内恢复未影响患者总生存。本组5例MM患者在预处理后均发生一过性AKI，且均为1级并在短期内恢复，提示肾功能不全MM患者在移植过程中的肾脏损伤相对轻微并可逆，不影响进行auto-HSCT。1例患者移植过程中肾功能损伤出现时间较晚（移植后19 d），且后续出现心功能衰竭，考虑为合并贫血、感染等综合原因所致，经处理后肾功能恢复至移植前，心功能好转，提示肾功能不全MM患者进行auto-HSCT仍存在一定风险，建议在有经验的移植中心开展。

综上，我们应用auto-HSCT治疗5例伴重度肾功不全MM患者获得了较好疗效且安全性良好，提示此类患者在有经验的移植中心行auto-HSCT是可行的。
